# Baishouwu Extract Suppresses the Development of Hepatocellular Carcinoma via TLR4/MyD88/NF-κB Pathway

**DOI:** 10.3389/fphar.2019.00389

**Published:** 2019-04-24

**Authors:** Yong-fang Ding, Zi-xuan Peng, Lan Ding, Yun-ru Peng

**Affiliations:** ^1^Department of Pharmacology and Toxicology, Jiangsu Province Academy of Traditional Chinese Medicine, Nanjing, China; ^2^Third College of Clinical Medicine, Xinjiang Medical University, Ürümqi, China; ^3^Department of Nephrology, Suzhou Wuzhong People’s Hospital, Suzhou, China

**Keywords:** Baishouwu extract, pretreatment, hepatocellular carcinoma, TLR4, inflammation-fibrosis-cancer axis

## Abstract

**Purpose:** The root of *Cynanchum auriculatum* Royle ex Wight, known as Baishouwu, has been widely used for a tonic supplement since ancient times. The current study was performed to explore the effect of Baishouwu extract on the development of experimental hepatocellular carcinoma (HCC) and the potential mechanism involved.

**Methods:** Rats were injected diethylnitrosamine (DEN) to initiate the multistep hepatocarcinogenesis. Animals were treated concurrently with Baishouwu extract given daily by oral gavage for 20 weeks to evaluate its protective effects. Time series sera and organ samples from each group were collected to evaluate the effect of Baishouwu extract on hepatic carcinogenesis.

**Results:** It was found that Baishouwu extract pretreatment successfully attenuated liver injury induced by DEN, as shown by decreased levels of serum biochemical indicators (AST, ALT, ALP, TP, and T-BIL). Administration of Baishouwu extract inhibited the fibrosis-related index in serum and live tissue, respectively from inflammation stage to HCC stage after DEN treatment. It significantly reduced the incidence and multiplicity of DEN-induced HCC development in a dose-dependent manner. Macroscopic and microscopic features suggested that pretreatment with Baishouwu extract for 20 weeks was effective in inhibiting DEN-induced inflammation, liver fibrosis, and HCC. Furthermore, TLR4 overexpression induced by DEN was decreased by Baishouwu extract, leading to the markedly down-regulated levels of MyD88, TRAF6, NF-κB p65, TGF-β1 and α-SMA in hepatitis, cirrhosis, and hepatocarcinoma.

**Conclusion:** In conclusion, Baishouwu extract exhibited potent effect on the development of HCC by altering TLR4/MyD88/ NF-κB signaling pathway in the sequence of hepatic inflammation-fibrosis-cancer, which provided novel insights into the mechanism of Baishouwu extract as a candidate for the pretreatment of HCC in the future.

## Introduction

Hepatocellular carcinoma (HCC) is the fifth most common cancer in the world and the third cause of cancer-related deaths (Jemal et al., 2011). Chronic inflammation, caused by chemical, biological and physical factors, is found to be related to certain human cancers. The effect of inflammation-fibrosis-cancer (IFC) axis acts as a bridge from inflammation to cancer, and therefore promotes inflamed liver evolving to fibrosis/cirrhosis and HCC (Lam et al., 2016; Ding et al., 2017). Upon exposure to risk factors like alcohol, viruses, parasitesand toxic substances, hepatic injury resulted in the degeneration and inflammation, leading to chronic liver diseases, which may further progress to different stages of fibrosis, cirrhosis, and HCC. HCC is the final stage of this process.

Traditional Chinese medicines contain natural active constituents, which demonstrate remarkable antitumor effect with little untoward effects. *Cynanchum auriculatum* Royle ex Wight is widely distributed in China. The root of this plant, known as “Baishouwu,” has been used for thousands of years as a tonic supplement. It is wildly used to replenish the liver and kidney, enrich vital essence and blood, strengthen the bones and muscles, clear away toxins, and prolong life (Liu and Ma, Song Dynasty). Modern studies indicated that the extract of this herb possessed various pharmacological activities, such as hepatoprotection, immune enhancement, antiaging, and antitumor (Zhang et al., 2015). Phytochemical and pharmacological studies have demonstrated that C-21 steroidal glycosides are the major active components of *Baishouwu* (Zhang et al., 2000; Wang et al., 2007; Peng et al., 2008a). Recently, C-21 steroidal glycosides are of considerable interest because of their bioactivities, including prevention and therapy of chronic hepatitis (Yin et al., 2007), hepatic fibrosis (Lv et al., 2009), and liver cancer (Wang et al., 2007, 2014). And our early studies showed that C-21 steroidal glycosides inhibited the growth of human hepatoma cell lines SMMC7721 and HepG2 (Peng et al., 2011; Peng and Ding, 2015]. Based on the literature and our studies, the C-21 steroidal glycosides were commonly accepted as the most important active ingredients of *Baishouwu* and could be considered as markers for the quality control. Hence, the C-21 steroidal glycosides constituents of Baishouwu extract have been identified and characterized in our following studies (Wang et al., 2017).

However, to date, limited information is known regarding the therapeutic effects of C-21 steroidal glycosides on HCC as well as their underlying mechanisms about IFC axis. In the present study, we aim to reveal the prevention of Baishouwu on diethylnitrosamine (DEN) -induced HCC model in rats at various stages of the disease’s progression, from hepatic inflammation to cancer.

## Materials and Methods

### Experimental Animals

Male Sprague-Dawley rats, weighing 150–180 g, were purchased from S.L.A.C. Laboratory Animal Co., Ltd. (Shanghai, China). Care of the animals used in this study was conducted according to the *Guide for the care and Use of Laboratory Animals* published by the U.S. National Institutes of Health (publication no. 85-23, revised 1996). Rats were housed under controlled temperature (22 ± 2°C) and relative humidity (40–70%) with a 12 h light/dark cycle, and allowed free access to standard rat-chow diet and tap water. All the experimental protocols were approved by our Academy Animal Experimental Ethical Committee.

### Extract Preparation

**Figure 1:**
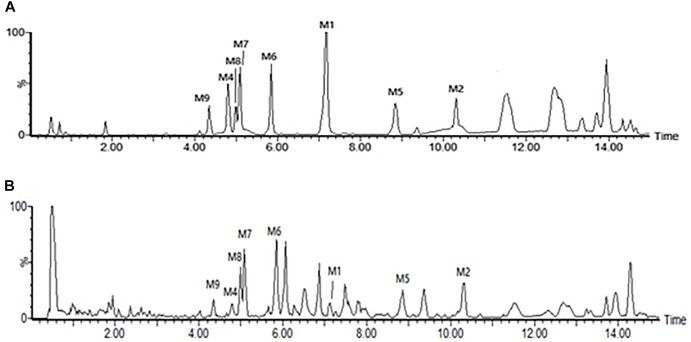
Total ion chromatograms in the negative mode of the reference compounds and the sample of Baishouwu extract. **(A)** Reference compounds and **(B)** Sample of Baishouwu extract. M1, caudatin 2,6-dideoxy-3-O-methy-β-D-cymaroseglycoside; M2, wilfoside C1N; M4, caudatin; M5, wilfoside K1N; M6, wilfoside C1G; M7, cynauricuoside A; M8, cynauricuoside C; M9, auriculoside IV.

The root tubers of *C. auriculatum* Royle ex Wight were collected from Binhai County, Jiangsu Province, China. The plant was authenticated by Prof. Shi-Hui Qian (Jiangsu Province Academy of Traditional Chinese Medicine). The voucher specimen (No. WFC-20151225) was deposited in the herbarium of this academy. The root tubers were cut into small pieces and extracted with boiling 95% ethanol (1:10) two times, each for 2 h. The ethanol extract was evaporated in *vacuo* and further extracted by chloroform and ethyl acetate. The two fractions were merged together and analyzed for major components. As detected according to the vanillin-vitriol colorimetric method, C-21 steroidal glycosides were the main components of Baishouwu extract and the total glycosides content was 52.89% (Wang et al., 2017). In order to identify the main chemical components of Baishouwu extract, an Ultra High-Performance Liquid Chromatography/Quadrupole-Time-of-Flight-Mass Spectrometry (UHPLC-Q-TOF-MS) method was developed. An Acquity UHPLC BEH C_18_ column (2.1 mm × 100 mm, 1.7 μm) was used for separations. The mobile phase was composed of (A) water (0.1% (v/v) formic acid) and (B) acetonitrile, and a linear gradient elution was used. It was revealed that Baishouwu extract mainly contained eight C-21 steroidal glycoside components including caudatin 2,6-dideoxy-3-O-methy-β-D-cymaroseglycoside, wilfoside C1N, caudatin, wilfoside K1N, wilfoside C1G, cynauricuoside A, cynauricuoside C, and auriculoside IV. The chemical structures were presented in our previous studies (Wang et al., 2017). The total ion chromatograms of the eight compounds are presented in Figure 1.

### Experimental Design

**Figure 2:**
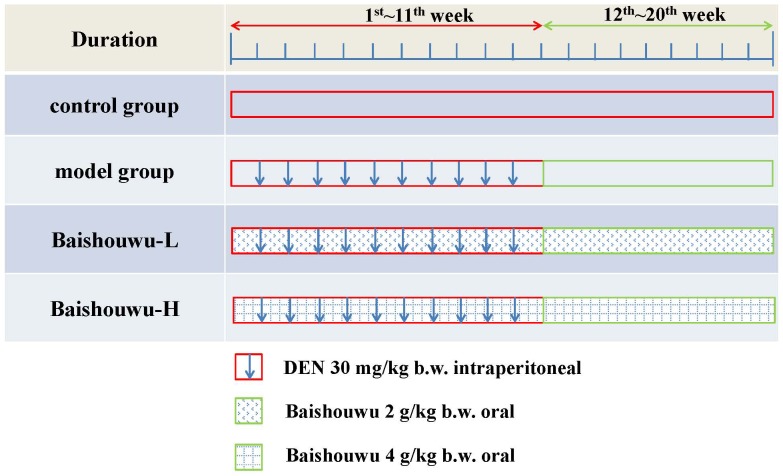
Experimental schedule.

Treatment schedules are illustrated in Figure 2. The rats were randomly divided into 4 groups. Control group was intraperitoneally injected with 0.9% normal saline solution twice a week. Rats in model group (DEN-bearing non-treated group) were given intraperitoneal injection of DEN (Sigma Aldrich, St. Louis, MO, United States) at a dose of 30 mg/kg body weight (b.w.) on the same day as control group for 11 weeks (Peng and Ding, 2015; Zeng et al., 2015; Ding et al., 2017). Rats in Baishouwu-H and Baishouwu-L groups were treated with DEN as in model group and daily orally fed with Baishouwu extract (4 g/kg b.w and 2 g/kg b.w, respectively) until 20 weeks.

Body weight of the animals was recorded once every 2 weeks throughout the study. To monitor the progress of stepwise hepatocarcinogenesis, according to our previous study, a time-serial sera set was collected at the end of 6th week (inflammation stage), 10th week (fibrosis stage), and 20th week (HCC stage). To examine the preventive effects of Baishouwu extract on HCC about IFC axis, 8 rats from every group were sacrificed by diethyl ether at 6th week, 10th week, and 20th week. Livers were removed and weighed. The tissues were cut and fixed in 10% formalin for histopathology and immunohistochemiacal examinations. The remaining portions were frozen and stored at -80°C until analysis.

### Serum Biochemical Indicators Levels and Hydroxyproline Contents Measurement

Serum aspartate transaminase (AST), alanine aminotransferase (ALT), alkaline phosphatase (ALP), total protein (TP), albumin (ALB), and total bilirubin (T-BIL) levels were measured using an automatic analyzer (C8000 Roche; Hoffmann-La Roche Inc., Switzerland). Serum levels of tumor necrosis factor-α (TNF-α) and interleukin 6 (IL-6) were measured using ELISA kits (R&D Systems, Inc.), according to the manufacturer’s instructions.

The hydroxyproline (Hyp) contents in whole liver specimens were quantified using ultraviolet spectrophotometry as previously reported (Ding et al., 2013). Hyp contents were expressed as μg/gram of livers wet weights.

### Morphometric Evaluation

Hepatic tissues of experimental groups of rats were examined morphologically for visible neoplastic nodules when the rats were sacrificed at the end of the study. The nodules were easily recognized and distinguished from the surrounding reddish-brown liver parenchyma. The nodules with a diameter of 2 mm or more in each rat were counted by two independent investigators. The incidence was expressed as percentage of rats with tumors. The multiplicity as average hepatocellular neoplasm number per rat was calculated (Ansil et al., 2014; Rui et al., 2014).

### Histologic Examination

Maximum sagittal section of each liver lobe was used for histopathological examination. Tissues were fixed in formaldehyde neutral buffer solution and embedded in paraffin blocks. 4 μm-thick sections were stained with hematoxylin-eosin (HE) for cell morphometry and with collagen-specific Masson’s trichrome for detection of fibrosis.

The degrees of liver necroinflammation were assessed against the METAVIR necroinflammatory activity score from A0–A3 (A0, no activity; A1, mild activity; A2, moderate activity; and A3, severe activity). The degrees of liver fibrosis were assessed using the METAVIR fibrosis score range F0-F4 (F0, no fibrosis; F1, portal fibrosis without septa; F2, portal fibrosis with rare septa; F3, numerous septa without cirrhosis; and F4, cirrhosis) (Bedossa and Poynard, 1996; Praneenararat et al., 2014). All sections were examined microscopically (200 × magnification) on an Axioskop 2 microscope (Carl Zeiss, Germany) by two blinded independent investigators.

### Immunohistochemical Analyses

Immunohistochemistry was employed to assess NF-κB expression in the rat liver tissue. The detection of NF-κB p65 was performed using paraffin-embedded sections. After deparaffinization and rehydration, sections were soaked in 3% H_2_O_2_ and skim milk to block endogenous peroxidase activity and non-specific protein binding, respectively. Samples were then incubated with anti-NF-κB p65 antibody (1:500, ab16502, Abcam) to detect bound antibodies. After staining, the sections were counterstained with hematoxylin for microscopy analyses. All sections were examined by light microscopy (Axioskop 2 microscope, Carl Zeiss, Germany). Five fields (200 × magnification) were randomly selected in each sample for analysis. Positive NF-κB signals appeared brown. The percentage of positive area was determined using Image-Pro Plus software 6.0 (Media Cybernetics Inc., Baltimore, MD, United States). The image analysis was conducted by pathologists blinded to the treatments.

### Western Blotting Analysis

Proteins were extracted from liver tissues randomly from three individual rats in each group using RIPA. Samples were separated by SDS-PAGE and transferred to PVDF membrane. Subsequently, membranes were blocked and probed with primary antibodies followed by secondary horseradish peroxidase-conjugated antibody. Immunolabeled proteins were detected by incubation with ECL substrate and the gray density was measured. Target proteins levels were normalized against the level of β-actin or Lamin B1. The following antibodies were used: TLR4 (1:1000, ab22048, Abcam, United Kingdom), MyD88 (1:1000, #4283, Cell Signaling Technology, United States), TRAF6 (1:800, sc-7221, Santa Cruz, CA, United States), NF-κB p65 (1:2000, ab16502, Abcam, United Kingdom), TGF-β_1_ (1:2000, ab25121, Abcam, United Kingdom), α-SMA (1:1000, ab5694, Abcam, United Kingdom), β-actin (1:1000, sc-47778, Santa Cruz, CA, United States), and Lamin B1 (1:10000, ab16048, Abcam, United Kingdom).

### RNA Extraction and Quantitative Real-Time PCR Detection

Total RNA was extracted from frozen liver tissues randomly from three individual rats in each group using Trizol reagent. Gene-specific prime sequences were designed using Primer 5.0 software and custom-synthesized by GenScript Inc., Nanjing, China. Real-time PCR samples were prepared using the SYBR premix EX Taq Kit (TaKaRs, Dalian, China) and amplification was performed on a LightCycler 480 SYBR Green I Master device (Roche Applied Science, Basel, Switzerland). GAPDH gene was used as an internal control. The relative expression levels of the target genes were calculated by the 2^-ΔΔCt^ method. The primer sequences utilized are shown in Table 1.

**Table 1:** Primer sequences of target genes.

Gene	Forward primer	Reverse primer
Collagen I	5-CTGCTGGTCCTAAGGGAGAG-3	5-GACAGCACCATCGTTACCAC-3
Collagen III	5-TCCTGGATACCAAGGTCCTC-3	5-GACCAATAGCACCAGGAGGT-3
TLR4	5-GATTGCTCAGACATGGCAGT-3	5-CCCACTCGAGGTAGGTGTTT-3
MyD88	5-TGGTGGTTGTTTCTGACGAT-3	5-GATCAGTCGCTTCTGTTGGA-3
TRAF6	5-GACATTCATGCACCTGGAAG-3	5-CATGTCAAAGCGGGTAGAGA-3
GAPDH	5-GGCCTTCCGTGTTCCTACC-3	5-CGCCTGCTTCACCACCTTC-3
α-SMA	5-CACGCGAAGCTCGTTATAGA-3	5-GGGATCCTGACCCTGAAGTA-3
TGF-β_1_	5-CTTGCCCTCTACAACCAACA-3	5-CTTGCGACCCACGTAGTAGA-3
NF-κB p65	5-GGTTTGAGACATCCCTGCTT-3	5-TATGGCTGAGGTCTGGTCTG-3

### Statistical Analysis

Data were expressed as mean ± standard deviation (SD). All statistical analyses were performed using the statistical package for social science (SPSS, Version 11.5, SPSS Inc., Chicago, IL, United States). Experimental and control groups were compared by one-way ANOVA. The incidences of hepatocellular neoplasm were analyzed by Chi-square test. The Kruskal-Wallist test was used to compare the METAVIR score. Statistical significance was set at *p*-value < 0.05.

## Results

### Effect of Baishouwu Extract on Body and Liver Weights

**Figure 3:**
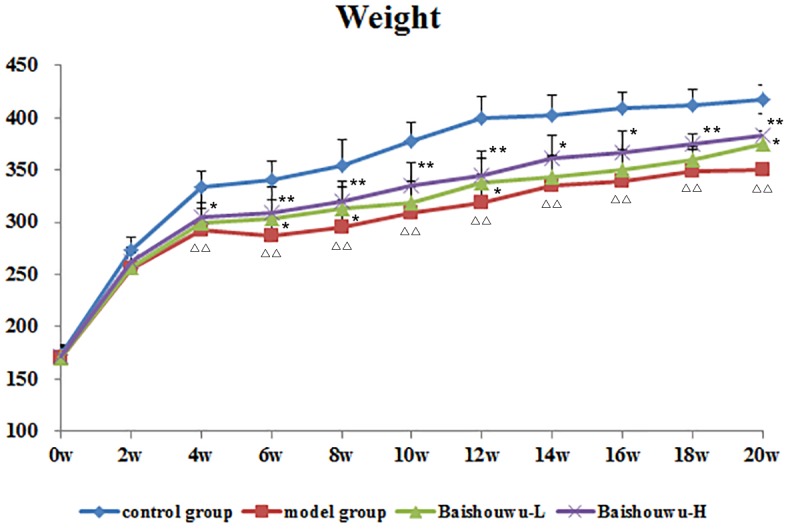
Effect of Baishouwu extract on growth curve of rats treated with DEN. Results are expressed as mean ± SD (1st∼6th week *n* = 24, 7th∼10th week *n* = 16, 11th∼20th *n* = 8, respectively). ^ΔΔ^*P* < 0.01 vs. control group, ^∗∗^*P* < 0.01 vs. model group.

Average body weights of different groups at various time points are shown in Figure 3. Animals in model group receiving 30 mg/kg of DEN exhibited slowly increased weight and the body weights were lower compared to control group. Body weights in Baishouwu-H and Baishouwu-L groups were higher than that in model group after the 20-week experimental period.

Relative liver weights of various groups are presented in Table 2. Relative liver weight were increased in model group induced by DEN than that in control group (*P* < 0.01). Liver to body weight ratios were significantly or tended to be lower in Baishouwu extract pretreated groups compared to the model group (*P* < 0.05∼0.01), respectively.

**Table 2:** Effect of Baishouwu extract on liver weights of rats treated with diethylnitrosamine.

Group	*n*	6th week	10th week	20th week
		Relative	Relative	Relative
		(/100 g b.w.)	(/100 g b.w.)	(/100 g b.w.)
Control group	8	1.972 ± 0.221	2.388 ± 0.2424	2.515 ± 0.280
Model group	8	4.224 ± 0.663^ΔΔ^	6.621 ± 1.25^ΔΔ^	8.430 ± 0.773^ΔΔ^
Baishouwu-L	8	3.707 ± 0.566	5.464 ± 0.74^∗^	6.612 ± 0.524^∗∗^
Baishouwu-H	8	3.405 ± 0.230^∗∗^	4.284 ± 0.332^∗∗^	4.568 ± 0.615^∗∗^

*Relative liver weight = [Liver weight (g)/Body weight (b.w., g) ^∗^100]. Values are expressed as the mean ± SD. ^ΔΔ^*P* < 0.01 vs. control group, ^∗^*P* < 0.05 vs. model group, ^∗∗^*P* < 0.01 vs. model group*.

### Baishouwu Extract Improves Liver Functions

**Figure 4:**
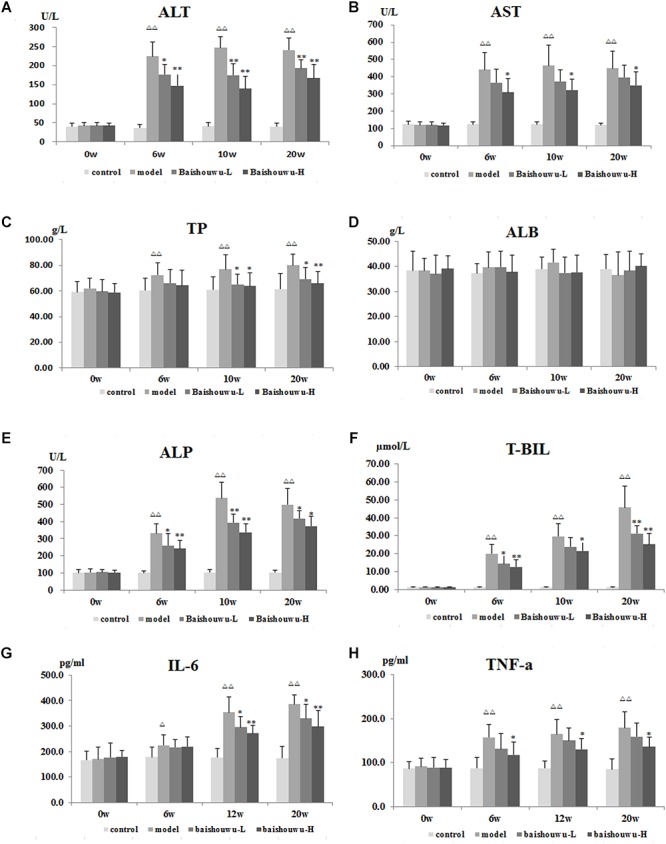
Effect of Baishouwu extract on serum biochemical indicators induced by DEN. Serum levels of **(A)** ALT, **(B)** AST, **(C)** TP, **(D)** ALB, **(E)** ALP, **(F)** T-BIL, **(G)** IL-6, and **(H)** TNF-α. Results are expressed as mean ± SD. (*n* = 8). ^ΔΔ^*P* < 0.01 vs. control group, ^∗^*P* < 0.05, ^∗∗^*P* < 0.01 vs. model group.

As shown in Figure 4A–F, serum biochemical analyses were performed to determine hepatic function. Both serum levels of ALT and AST were significantly increased in DEN-treated group which were prevented by Baishouwu extract from inflammation stage (week 6) to HCC stage (week 20). In parallel, there was a decrease in serum concentration of TP in Baishouwu extract group at week 10 and week 20 (*P* < 0.05∼0.01, compared to the model group). But no significant changes of serum ALB levels were found between all groups. Serum level of ALP was higher in the DEN-treated group compared to the control group. Baishouwu extract pre-administration resulted in suppression of ALP levels in the blood serum in a dose-dependent manner as compared with the model group. Serum T-BIL level was also significantly higher in the model group than that in the control. Pretreatment with Baishouwu extract dramatically reduced the T-BIL levels from week 6 to week 20 (*P* < 0.05∼0.01, compared to the model group).

The serum levels of inflammatory factors, such as IL-6 and TNF-α, were analyzed subsequently. Serum IL-6 levels in the model group were obviously increased in inflammation, liver fibrosis and HCC and they were suppressed by pre-administration of Baishouwu extract at week 12 and week 20 (Figure 4G). Serum TNF-α levels were higher in the model group than in control group from week 6 to week 20 (*P* < 0.01). Baishouwu extract pretreated group with high dose displayed a decrease in comparison to that from the model group (*P* < 0.05; Figure 4H).

### Baishouwu Extract Inhibits the Expression of Collagen

**Figure 5:**
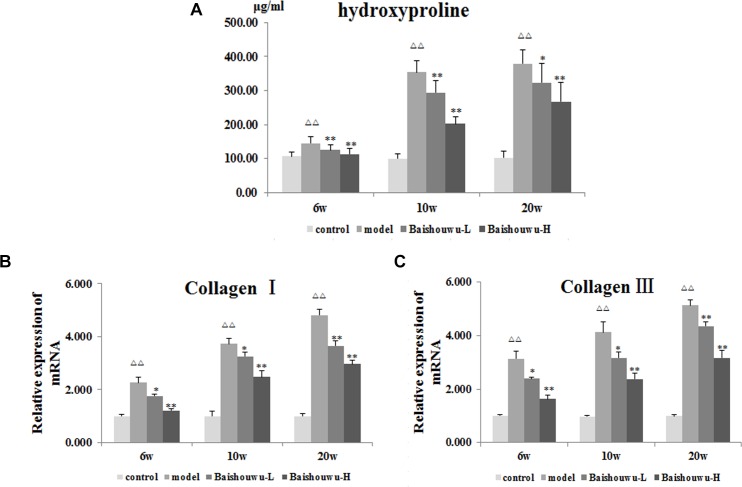
Effect of Baishouwu extract on fibrosis related indicators induced by DEN. Tissue levels of **(A)** hydroxyproline, **(B)** Collagen I, and **(C)** Collagen III. Results are expressed as mean ± SD (*n* = 8). ^ΔΔ^*P* < 0.01 vs. control group, ^∗^*P* < 0.05, ^∗∗^*P* < 0.01 vs. model group.

As a biomarker of total collagen content, Hyp was detected. Data was showed in Figure 5A. DEN administration markedly increased the liver Hyp content in rats. Compared with the model group, the elevated Hyp contents were significantly suppressed by the pretreatment of Baishouwu extract at both dose levels throughout the experiment period (*P* < 0.05∼0.01).

A gradual increase in mRNA expression of Collagen I and Collagen III was observed after administration of DEN compared with the control group (*P* < 0.01 Figure 5B,C). Prereatment with Baishouwu extract prevented the increase in Collagen I and Collagen III mRNA levels in inflammation, fibrosis, and HCC stages. This result was consistent with the observation in Hyp.

### Baishouwu Extract Reduces the Hepatoma Incidence and Multiplicity Induced by DEN

**Figure 6:**
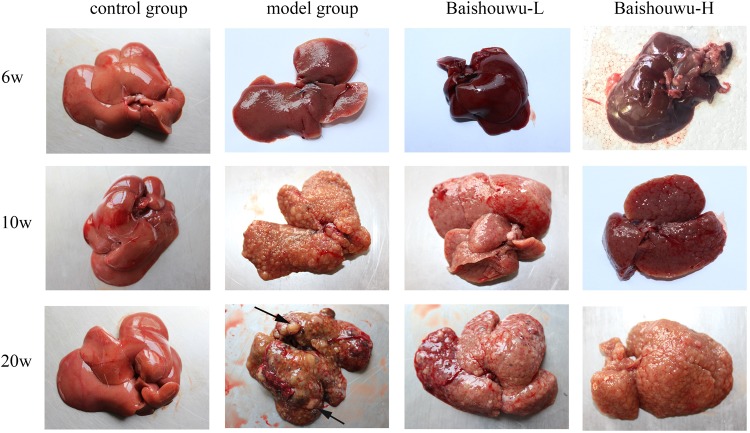
Effect of Baishouwu extract on macroscopic features of livers in DEN-treated rats. Representative photographs of the livers from each group at the stages of inflammation (6 weeks), fibrosis (10 weeks), and HCC (20 weeks). Arrows indicate the representative tumors.

Morphological changes were observed in the livers of different groups at different stages (inflammation, fibrosis, and HCC stages). As expected, livers from the control group of rats were normal in appearance, without any morphological changes. The surface of the liver of the control group was brown, soft in texture and smooth, with an evident gloss. Macroscopically, the appearance of the liver was not evidently abnormal in the model group in the inflammation stage at 1–6 weeks (Figure 6). Following DEN exposure, the livers with dark color and granulose appearance in the model group were slightly bigger and harder than those of control-rats in fibrosis stage (Figure 6). The livers of rats treated with Baishouwu extract showed decreased hepatic fibrosis progression. As shown in Figure 6, the rats at week 20 post-DEN exposure were observed white nodules, indicating that HCC had developed by this stage. Nodules were easily recognized and distinguished from the surrounding non-nodular reddish-brown liver parenchyma at the end of week 20. The livers in the model group were rough and nodular, with uniform micronodules (<2 mm) and macronodules (≥2 mm) throughout. Pretreatment with Baishouwu extract remarkably enhanced the recovery of DEN-induced liver structure damage (Figure 6). The nodule incidence was 100% in model rats, while the incidence decreased to 87.5% in Baishouwu extract group (4 g/kg). Furthermore, the number of liver nodules in Baishouwu-pretreated rats was less as compared to the model rats, with a significant difference at week 20 (*p* < 0.01; Table 3). So the incidence and multiplicity of HCC development in Baishouwu-pretreated rats were significantly lower than that in the model rats.

**Table 3:** Effect of Baishouwu extract on incidence and multiplicity of tumor in diethylnitrosamine induced hepatoma rats.

Group	*n*	Incidence	Multiplicity
Control group	8	0	0 ± 0
Model group	8	8/8	155.4 ± 34.2^ΔΔ^
Baishouwu-L	8	8/8	70.8 ± 12.5^∗∗^
Baishouwu-H	8	7/8	50.1 ± 17.4^∗∗^

*Values are expressed as the mean ± S.D. ^ΔΔ^*P* < 0.01 vs. control group, ^∗∗^*P* < 0.01 vs. model group*.

### Baishouwu Extract Alleviates Hepatic Pathological Changes

**Figure 7:**
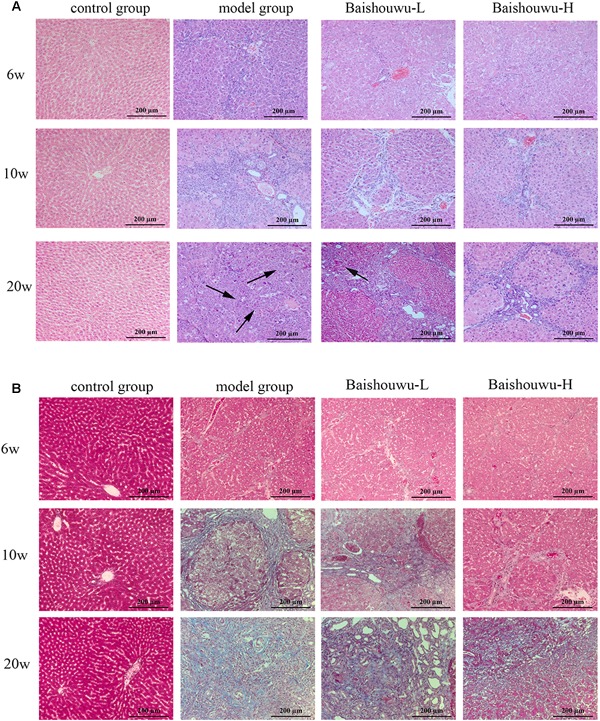
Effect of Baishouwu extract on the liver histological changes in the DEN-treated rats. Representative photomicrographs of HE **(A)** and Masson **(B)** staining of the livers in the DEN-treated rats from each group at the stages of inflammation (6 weeks), fibrosis (10 weeks), and HCC (20 weeks). Arrows indicate the representative tumors. Original magnification: 200×.

The histopathological changes observed in the livers of control and experimental animals are represented in Figure 7. Livers from control rats showed normal liver histology with no signs of liver injury manifested as normal hepatic lobules and central vein (Figure 7). Exposure to DEN resulted in a sequence of lesions that evolved over time, from inflammation observed at 6th week, fibrosis observed at 10th week, to the HCC observed at 20th week. Six weeks after DEN injection (inflammation stage), liver sections showed evidence features of chronic inflammatory infiltrates with diffuse ballooning degeneration, dilated lymph vessels, and proliferating bile ducts in the portal area (Figure 7). Liver from rats pretreated with Baishouwu extract showed no significant hepatic injury manifested as minimal vascular congestion and focal lymphoplasmacytic infiltrates (Figure 7), which correspond to the METAVIR score (Table 4).

**Table 4:** Effect of Baishouwu extract on the distribution of inflammatory features in the diethylnitrosamine treated rats assessed by METAVIR score.

Time	Group	A0	A1	A2	A3	*P* value (vs. model)
Inflammation stage (6 weeks)	Control	8 (100%)	0 (0%)	0 (0%)	0 (0%)	0.001
	Model	0 (0%)	0 (0%)	0 (0%)	8 (100%)	
	Baishouwu-L	0 (0%)	3 (37.5%)	3 (37.5%)	2 (25.0%)	0.001
	Baishouwu-H	0 (0%)	4 (50.0%)	4 (50.0%)	0 (0%)	0.001
Fibrosis stage (10 weeks)	Control	8 (100%)	0 (0%)	0 (0%)	0 (0%)	0.001
	Model	0 (0%)	0 (0%)	0 (0%)	8 (100%)	
	Baishouwu-L	0 (0%)	2 (25.0%)	2 (25.0%)	4 (50.0%)	0.001
	Baishouwu-H	0 (0%)	3 (37.5%)	3 (37.5%)	2 (25.0%)	0.001
HCC stage (20 weeks)	Control	8 (100%)	0 (0%)	0 (0%)	0 (0%)	0.001
	Model	0 (0%)	0 (0%)	0 (0%)	8 (100%)	
	Baishouwu-L	0 (0%)	0 (0%)	3 (37.5%)	5 (62.5%)	0.001
	Baishouwu-H	0 (0%)	1 (12.5%)	4 (50.0%)	3 (37.5%)	0.001

*Compared to model group; calculated using the Chi-Square test. METRAVIR inflammation stages: A0, no activity; A1, mild activity; A2, moderate activity; A3, severe activity*.

After 10 weeks of DEN injury (fibrosis stage), the animals exhibited fibrosis, and many also had cirrhosis. As Figure 7 shown, lobules of livers in model group exhibited a disordered arrangement of hepatocytes and a pile of deposition of fibrous tissue. Lipid droplets, hydropic degeneration, necrosis, and regeneration of hepatocytes were found. According to the METAVIR liver fibrosis assessment scales, Baishouwu extract preadministration decreased the extent of liver fibrosis induced by DEN (Table 5). By preadministration of Baishouwu extract, the animal models had successfully developed the different stages of early liver fibrosis.

**Table 5:** Effect of Baishouwu extract on the distribution of fibrosis features in the diethylnitrosamine treated rats assessed by METAVIR score.

Time	Group	F0	F1	F2	F3	F4	*P* value (vs. model)
Inflammation stage (6 weeks)	Control	8 (100%)	0 (0%)	0 (0%)	0 (0%)	0 (0%)	0.001
	Model	0 (0%)	(0%)	8 (100%)	0 (0%)	0 (0%)	
	Baishouwu-L	0 (0%)	6 (75.0%)	2 (25.0%)	0 (0%)	0 (0%)	0.001
	Baishouwu-H	0 (0%)	8 (100%)	0 (0%)	0 (0%)	0 (0%)	0.001
Fibrosis stage (10 weeks)	Control	8 (100%)	0 (0%)	0 (0%)	0 (0%)	0 (0%)	0.001
	Model	0 (0%)	0 (0%)	0 (0%)	2 (25.0%)	6 (75.0%)	
	Baishouwu-L	0 (0%)	2 (25.0%)	2 (25.0%)	2 (25.0%)	2 (25.0%)	0.001
	Baishouwu-H	0 (0%)	4 (50.0%)	2 (25.0%)	1 (12.5%)	1 (12.5%)	0.001
HCC stage (20 weeks)	Control	8 (100%)	0 (0%)	0 (0%)	0 (0%)	0 (0%)	0.001
	Model	0 (0%)	0 (0%)	0 (0%)	0 (0%)	8 (100%)	
	Baishouwu-L	0 (0%)	0 (0%)	1 (12.5%)	3 (37.5%)	4 (50.0%)	0.001
	Baishouwu-H	0 (0%)	1 (12.5%)	2 (25.0%)	3 (37.5%)	2 (25.0%)	0.001

*Compared to model group; calculated using the Chi-Square test. METRAVIR fibrosis stages: F0, no fibrosis; F1, portal fibrosis without septa; F2, portal fibrosis with rare septa; F3, numerous septa without cirrhosis; F4, cirrhosis*.

After 20 weeks of DEN injection (HCC stage), the liver lesions of model group were classified as the high or middle differentiation of HCC. DEN alone (model group) showed loss of architecture, hepatic parenchyma with granular cytoplasm and cancerous focus with patchy necrosis (Figure 7). Liver nodules consisted of relatively giant hepatocytes with large nuclei and eosinophilic cytoplasm surrounded by fibrotic stroma. HCC features appeared as malignant hepatocytes, multinodular areas of necrosis, and nodules of necrotic malignant hepatocytes. DEN-bearing Baishouwu extract-pretreated groups showed moderately malignant with focal necrosis and less mitotic count (Figure 7).

### Baishouwu Extract Down-Regulates the Expression of NF-κB p65 by Immunohistochemistry

**Figure 8:**
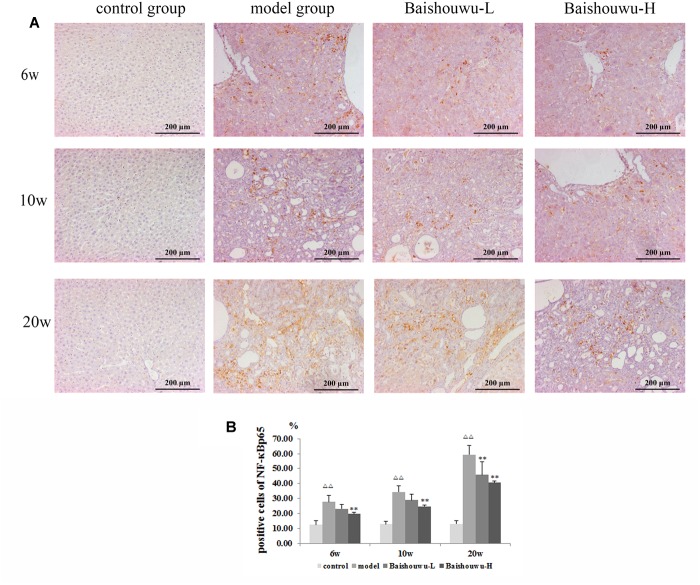
Effect of Baishouwu extract on the expression of NF-κB p65 in the livers of the DEN-treated rats. Representative photomicrographs of immunohistochemical analysis of NF-κB p65 in the livers developed in DEN-treated rats. **(A)** Representative liver tissues immunostained with anti- NF-κB p65 antibody in hepatitis, cirrhosis and hepatocarcinoma control at 6th week, 10th week, and 20th week (magnification: 200×). **(B)** The percentage of NF-κB p65 positive cells in control and experimental groups. Results are expressed as mean ± SD (*n* = 3). ^ΔΔ^*P* < 0.01 vs. control group, ^∗∗^*P* < 0.01 vs. model group.

NF-κB p65 positive staining was mainly located in the nucleus of the hepatic cells, stained as brown granules or dots. Its expression was estimated as the percentage of cells positively stained by the antibody. In normal control liver section there was a limited number of NF-κB p65 positive cells as shown in Figure 8A. Rats which received DEN showed a significant increase compared with the control group. The positive expression rates of the NF-κB p65 protein in the inflammation, fibrosis and HCC tissues were 27.9, 34.5, and 59.4%, respectively. The expression of the NF-κB p65 protein in HCC stage was significantly higher than those in the inflammation and fibrosis. On the other hand, Baishouwu extract-pretreated rats showed considerable reduction compared with the model group (*p* < 0.05∼0.01; Figure 8B) in inflammation, fibrosis and HCC stages.

### Baishouwu Extract Pretreatment Is Associated With Down-Regulated TLR4/MyD88/NF-κB Signaling Pathway

**Figure 9:**
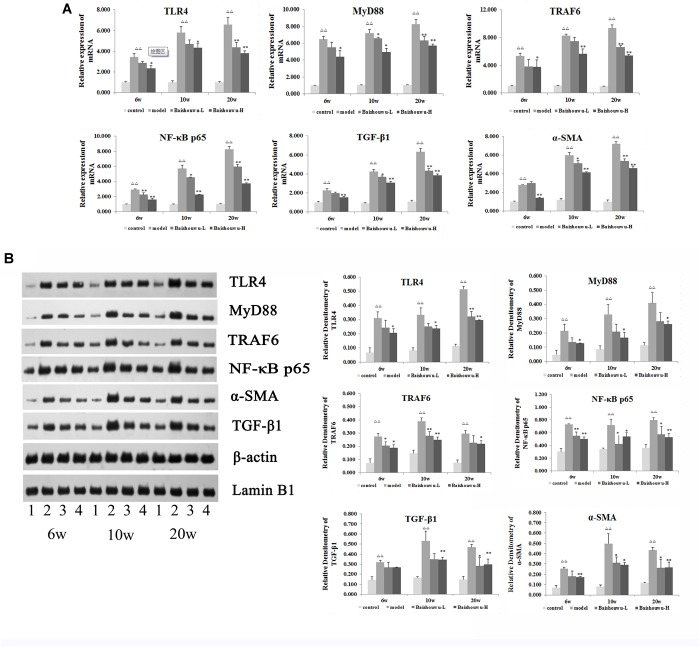
Effect of Baishouwu extract on the TLR4/MyD88/NF-κB signaling pathway. The expression levels of TLR4, MyD88, TRAF6, NF-κB p65, TGF-β_1_, and α-SMA were detected by Real-time PCR **(A)** and western blot **(B)**: (1) control group, (2) model group, (3) Baishouwu extract-L group, and (4) Baishouwu extract-H group. Results are expressed as mean ± SD (*n* = 3). ^ΔΔ^*P* < 0.01 vs. control group, ^∗^*P* < 0.05, ^∗∗^*P* < 0.01 vs. model group.

TLR4 is an important mediator of the inflammatory response to infection and plays a role in the development and progression of HCC (Wang et al., 2015). TLR4 drives myofibroblast activation and fibrogenesis in HCC and TLR4-dependent modulation of TGF-β signaling provides a link between proinflammatory and profibrogenic signals through the MyD88- NF-κB pathway (Seki et al., 2007). TLR signaling involves the recruitment of MyD88 adapter protein and final activation of NF-κB. To confirm the hepatoprotective effect of Baishouwu extract, the protein and mRNA expression levels of TLR4, MyD88, TRAF6, NF-κBp65, TGF-β_1_, and α-SMA in the livers were examined at inflammation, fibrosis and HCC stages in DEN-induced HCC model. After DEN treatment, the mRNA levels of TLR4, MyD88, TRAF6, and NF-κB p65 were significantly increased compared to the control group from week 6 to week 20 (Figure 9A), which were demonstrated by western blotting (Figure 9B). All of these results suggested that TLR4/MyD88/NF-κB signaling pathway was activated in the liver of DEN-treated rats (Figure 9). However, these elevations were reversed by the pretreatment with Baishouwu extract (Figure 9). At the same time, the changes of TGF-β_1_ and α-SMA after Baishouwu extract pretreatment for 6 weeks, 10 weeks, and 20 weeks were consistent with the changes of TLR4 (Figure 9). The results indicated that Baishouwu extract may inhibit hepatic inflammation, fibrosis, and HCC by inhibiting TLR4/MyD88/NF-κB signaling pathway.

## Discussion

As the main active components of Baishouwu, C-21 steroidal glycosides have shown to possess anticancer activity including modulating cell cycle and apoptotic signaling, inhibiting invasion, and metastatic potential in cancer cells (Shan et al., 2005; Fei et al., 2012; Wang et al., 2017). Our previous studies found that C-21 steroidal glycosides could inhibit the proliferation of human hepatoma cell by inducing cell apoptosis through caspase-3 activation (Peng et al., 2008b, Peng et al., 2011). It was also reported that a C-21 steroidal glycoside from Baishouwu exhibited anti-tumor activity against human gastric cancer cells by inducing G1 phase cell cycle arrest and capase-dependent apoptosis cascades (Wang et al., 2013). Recent studies have demonstrated that ERK/JNK/MAPK pathways were involved in C-21 steroidal glycosides induced tumor cells apoptosis (Fei et al., 2012; Fu et al., 2015). It has been widely accepted that Baishouwu extract, especially the C-21 steroidal glycosides, is effective in anticancer, but the potential molecular mechanism remains largely unknown. In the present study, a rat model with HCC was developed to evaluate the effect of Baishouwu extract on different stages of DEN-induced hepatic IFC sequence by influencing the inflammatory signaling pathway.

Chronic inflammation of the liver is a well-recognized risk factor for carcinogenesis, the molecular link between inflammation, hepatic fibrogenesis, and HCC remains elusive. The liver is the main site of inflammatory response to intestine-derived bacterial products crossing the intestinal barrier. Recently, several studies implicated that toll-like receptors (TLRs) and their proinflammatory mediators may be of the host inflammatory response to infection and plays a role in the human hepatic IFC axis (Abastado, 2012; Soares et al., 2012; Li W. et al., 2015). TLRs represent one important receptor family which enables the innate immune system to immediately react to infections. They contribute to adaptive immune reactions and the regulation of sterile inflammation and tissue regeneration as well as carcinogenesis (O’Neill et al., 2013; Roh and Seki, 2013). Of the TLRs, TLR4 responsibles for detecting Gram-negative bacteria including lipopolysaccharide (LPS), whereas TLR2 identifies components of Gram-positive bacteria such as peptidoglycan (Kawai and Akira, 2010). There are several types of TLR-expressing cells in the liver, including hepatocytes, Kupffer cells (Mcdonald et al., 2013), stellate cells (Zhu et al., 2012), sinusoidal endothelial cells (Suzuki et al., 2016), and biliary epithelial cells (Kim et al., 2016). Of these cells, the role of hepatic stellate cells (HSC) has been extensively investigated in the development of liver inflammation, fibrosis and subsequent tumor via TLR4 (Dapito et al., 2012). TLR4-mediated inflammation is critical in both host defenses against invading pathogens and for physiological responses to inflammatory stimuli. Ligation of TLR4 also markedly promotes liver fibrosis (Aoyama et al., 2010). Similarly, TLR4 ligation by LPS derived from selected intestinal microbiota promotes hepatocellular carcinogenesis (Dapito et al., 2012). Overall, hepatic expression of TLR4 is increased in chronic hepatitis and cirrhosis and is maintained in hepatocarcinoma.

It had been suggested that DEN-induced liver injury was accompanied by elevation of plasma LPS level, and then LPS can transiently exaggerated DEN-induced liver damage (Yu et al., 2010). After binding to TLR4, two critical intracellular signaling pathways are triggered, including the myeloid differentiation primary response 88 (MyD88) dependent and MyD88-independent signaling cascades (Pang et al., 2018). The MyD88-dependent signal transduction activates NF-κB through activation of its inhibitory protein IκBα, which allows NF-κB nuclear translocation and controls the expression of a multitude of proinflammatory cytokines and other immune-related genes, such as TNF-α, IL-1, IL-1β, IL-6, and IL-12 (Shi et al., 2013; Li X. et al., 2015). In the parallel MyD88-independent, TIR-domain-containing adaptor-inducing interferon-β (TRIF) pathway, TRIF associates with TRAF3 and TRAF6 to activate tank-binding kinase-1 (TBK1) and I-kappa-B kinase epsilon (IKKi), which results in the activation of interferon regulatory transcription factor 3 (IRF3) and IRF7 (Bagchi et al., 2007; Yu et al., 2010). Activated IRF3 and IRF7 drive transcription of interferon-α (IFN-α), IFN-β and IFN-responsive genes. It is reasonable that both of TLR4 signals can induce liver inflammation, promote fibrosis, and aggravate progression toward malignancies in TLR4-dependent cancers such as HCC (Demaria et al., 2010; Szabo and Petrasek, 2015). In the present work, the antinflammatory capability of Baishouwu extract mainly resulted in decreased levels of MyD88, TRAF6, NF-κB, IL-6 and TNF-α via the reduction of TLR4 expression in hepatitis, cirrhosis, and hepatocarcinoma stages of the HCC models induced by DEN. Thus, TLR4 signaling pathway was involved in the inhibition of proinflammatory cytokines expression by Baishouwu extract rich in C-21 steroidal glycosides. Therefore, these results supported a novel effect of C-21 steroidal glycosides on the antinflammatory ability, which suggested C-21 steroidal glycosides as a promising pretreatment agent for ameliorating the development of HCC.

We and others have previously shown that chronic hepatic inflammation was tightly linked to fibrosis and hepatocarcinogenesis (Wai et al., 2015; Ding et al., 2017), but the molecular link remains elusive. Our study has identified TLR4 signaling as a central mediator in liver tumor via TLR4-MyD88-NF-κB pathway. On the other hand, it is well-known that TGF-β pathway caused by activation of at least one of the TGF-β signaling components is an important risk factor for fibrogenesis and HCC in man and animal models (Yang et al., 1999; Kitisin et al., 2007). Activation of TLR4 enhances TGF-β signaling in the development of hepatic fibrosis. The lack of functional type I and type II TGF-β receptors and Smad3 results in extensive inflammation due to increased TLR4 expression (Yang et al., 1999; Lucas et al., 2000; Mccartneyfrancis et al., 2004). Studies on TLR4-deficient mice uncovered that TLR4 mediated the regulation of the TGF-β_1_ pseudoreceptor BAMBI (bone morphogenic protein and active membrane bound inhibitor) by TLR4-MyD88-NF-κB-dependent pathway, thus sensitizing HSCs to TGF-β_1_ signaling. HSCs were being activated and consequently increased proliferation and caused overexpression of α-SMA. The enhanced expression of α-SMA indicated that the invasion of numerous fibroblasts (Friedman, 2007; Seki et al., 2007). The cross talk between TLR4 and TGF-β pathway provides a link between proinflammatory and profibrogenic signals in HCC. Here, we demonstrated that TLR4, MyD88, TGF-β_1_ expressions, and NF-κB p65 nuclear translocation increased in model rats at 6th, 10th and 20th week, but Baishouwu extract pretreatment downregulated TLR4, MyD88, and TGF-β_1_ expressions and even suppressed downstream signaling through NF-κB p65 nuclear translocation. Moreover, the expression of α-SMA and collagen I and II, the marker of HSCs activation and the major components of extracellular matrix in fibrotic liver, were significantly elevated in the DEN-treated rats and were inhibited by Baishouwu extract. Taken together, our results suggest that Baishouwu extract maybe contribute to modulation of TGF-β signaling by TLR4-MyD88-NF-κB-dependent pathway in HCC, but the precise molecule mechanism of Baishouwu extract on the crosstalk between TLR4 and TGF-β pathway requires further investigation.

In conclusion, based on IFC axis, Baishouwu extract exhibited potent effect on the development of HCC, and their underlying mechanism might by associate with altering TLR4/MyD88/ NF-κB signaling pathway in the sequence of hepatic IFC. Our study provided novel insights into the mechanism of Baishouwu extract as a candidate for the pretreatment of HCC in the future.

## Ethics Statement

This study was carried out in accordance to the recommendation of *Guide for the Care and Use of Laboratory Animals* published by the US National Institutes of Health (Publication No. 85-23, revised 1996). The protocol was approved by the Animal Experimental Ethical Committee of Jiangsu Province Academy of Traditional Chinese Medicine.

## Author Contributions

All authors designed and carried out the experimental work, read, and approved the final manuscript. Z-xP analyzed the statistical data and interpretation the results. Y-fD and LD drafted and critically evaluated the manuscript.

## Conflict of Interest Statement

The authors declare that the research was conducted in the absence of any commercial or financial relationships that could be construed as a potential conflict of interest.
